# Poor weight gain and its predictors among preterm neonates admitted at Muhimbili National Hospital in Dar-es-salaam, Tanzania: a prospective cohort study

**DOI:** 10.1186/s12887-021-02971-y

**Published:** 2021-11-06

**Authors:** Victoria Paul Ndembo, Helga Naburi, Rodrick Kisenge, Germana H. Leyna, Candida Moshiro

**Affiliations:** 1 Department of Paediatrics and Child Health, Muhimbili National Hospital (MNH), P.O. Box 65000, Dar es Salaam, Tanzania; 2 Department of Paediatrics and Child Health, Muhimbili University of Health and Allied Sciences (MUHAS), Dar es Salaam, Tanzania; 3 Department of Epidemiology and Biostatistics, Muhimbili University of Health and Allied Sciences (MUHAS), Dar es Salaam, Tanzania

**Keywords:** Preterm, Poor weight gain, Feeding, Muhimbili

## Abstract

**Background:**

Preterm delivery is among the major public health problems worldwide and the leading cause of morbidity and mortality among neonates. Postnatal poor weight gain, which can contribute to mortality, can be influenced by feeding practices, medical complications and quality of care that is provided to these high-risk neonates. This study aimed to investigate the proportion and predictors of poor weight gain among preterm neonates at Muhimbili National Hospital (MNH), from September 2018 to February 2019.

**Methods:**

A hospital-based prospective cohort study involving preterm neonates with Gestation age (GA) < 37 weeks receiving care at MNH. Eligible preterm, were consecutively recruited at admission and followed up until discharge, death or end of neonatal period. Poor weight gain was defined as weight gain less than 15 g per kg per day. The risk factors associated with poor weight gain were evaluated. Predictors of poor weight gain were evaluated using a multivariate analysis. Results were considered statistically significant if P -value was < 0.05 and 95% confidence interval (CI) did not include 1.

**Results:**

A total of 227 preterm neonates < 37 weeks GA, with male to female ratio of 1:1.2 were enrolled in the study. The overall proportion of preterm with poor weight gain was 197/227 (86.8%). Proportion of poor weight gain among the early and late preterm babies, were 100/113 (88.5%) and 97/114 (85.1%) respectively. Predictors of poor weight gain were low level of maternal education (AOR = 2.58; 95%Cl: 1.02–6.53), cup feeding as the initial method of feeding (AOR = 8.65; 95%Cl: 1.59–16.24) and delayed initiation of the first feed more than 48 h (AOR = 10.06; 95%Cl: 4.14–24.43). A previous history of preterm delivery was protective against poor weight gain (AOR = 0.33; 95% Cl: 0.11–0.79).

**Conclusion and recommendation:**

Poor weight gain was a significant problem among preterm neonates receiving care at MNH. This can be addressed by emphasizing on early initiation of feed and tube feeding for neonates who are not able to breastfeed. Health education and counselling to mothers focusing on feeding practices as well as close supervision of feeding especially for mothers experiencing difficulties in feeding their preterm will potentially minimize risk of growth failure.

**Supplementary Information:**

The online version contains supplementary material available at 10.1186/s12887-021-02971-y.

## Background

The prevalence of preterm deliveries worldwide is higher especially in developing countries. This is associated with increased risk of low birth weight and poor health outcomes including poor growth and development. According to WHO 2018 report [1] preterm birth remains a significant public health concern, and it is the leading cause of neonatal mortality and morbidity.

The World Health Organization (WHO) in 2018, estimated that 15 million babies are born too early each year; and the burden is increasing in many low- and middle-income countries [1]. Studies in Tanzania have also reported a high prevalence of preterm birth between 10 and 16.7% [2].

Postnatal growth pattern in preterm and LBW neonates is often characterized by initial physiological weight loss in the first 7 days of life of approximately 7–15% of their birth weight. Thereafter recovery occurs with increase in body weight from around 10th to 21st day of life [3, 4]. However, differences in weight trends have been observed with respect to the age of the preterm, being more marked amongst early and extreme preterm as compared to late preterm [5].

Preterm babies can have feeding problems because the coordinated suck and swallow reflex is not yet fully developed [6], thus may require some degree of feeding support until they are mature and stable enough to feed exclusively by mouth. Furthermore, preterm babies are more likely to medical complications during postnatal period such as respiratory distress syndrome, apnoea, hypothermia, anaemia and sepsis, which further worsens their feeding problem [7]. Regardless of the cause poor nutrition puts them at risk for permanent detrimental effects including poor growth and development [8, 9]. Since, appropriate and adequate nutrition is essential for the growth and development of these infants, the WHO has recommended several feeding practices which can influence postnatal weight gain [10]. However, since most of preterm feeding depends on caretaker postnatal weight gain will be influenced by feeding knowledge and practices of the caretakers. Thus, close monitoring and supervision of mothers when feeding their preterm babies especially those with medical complications can serve as an early intervention that can influence better growth and development of these babies.

Despite the high burden of preterm births in Tanzania, there is paucity of data on the proportion and predictors of poor weight gain in preterm neonates. Understanding this will help develop and improve existing feeding guidelines for management of these babies in Tanzania and similar settings. Therefore, this study set to establish the predictors of poor weight gain among preterm neonates admitted in a neonatal unit at a tertiary hospital, Muhimbili in Dar es salaam, Tanzania.

## Methods

### Study design and area

This hospital-based prospective cohort study was carried out in neonatal and Kangaroo Mother Care (KMC) wards at Muhimbili National Hospital (MNH) from September 2018 to February 2019. The unit receives all premature and term babies born in the hospital and those referred from other hospitals. The unit is divided into three wards including a ward for term babies, a ward for unstable preterm babies and a Kangaroo Mother Care (KMC) ward for stable preterm and very low birth weight babies receiving KMC.

In our hospital the health care workers do recommend the feeding method based on GA and clinical condition of the baby. During the first feed mothers are taught by nurses how to feed by cup or NGT and subsequently mothers continues to feed their babies every 3 h as per hospital guidelines.

The hospital also runs a high-risk outpatient clinic twice a week for all preterm babies discharged from the newborn unit. In these clinics preterm are followed up weekly until end of neonatal period, and monthly until they reach a chorological age of 6 months and thereafter transitioned to nearby clinics.

### Study population and eligibility criteria

All preterm neonates (less than 37 weeks GA) corrected age admitted in neonatal at MNH during the study period were eligible for enrollment. We excluded all preterm who had major congenital anomalies interfering with feeding and syndrome babies as well as preterm admitted more than 48 h of age with no record of feeding or weight measurements.

Screening for eligibility of the preterm was done using gestational age estimation based on Ballard score and where results had not been documented within 24 h the default was LNMP, which was also crosschecked with the expected date of delivery documented on their antenatal cards. Only parents/caregivers of preterm neonates satisfying the inclusion criteria were interviewed upon obtaining an informed written consent.

Those preterm who were eligible and included in the study were followed up for the period of 4 weeks (neonatal period).

### Data collection

A structured questionnaire translated in local language (Kiswahili) was used to interview mothers, to obtain baseline characteristics of preterm and caretakers as well as information on feeding practices and caretaker’s knowledge. Knowledge was measured using 5 items developed based on questions adopted from WHO guidelines on optimal feeding of preterm. The questions asked about optimal duration of exclusive breastfeeding, preferable type of milk, preferable method of feeding, frequency of feeding and time to initiate first feed in a preterm [10].

Clinical characteristics of the study participants including comorbid conditions were extracted form patient files. Birth weight was extracted from the perinatal chart, as recorded in grams, and subsequent weights were measured by a researcher and research assistants on daily basis from 24 h post admission until discharge, death or end of neonatal period (4 weeks). The weight was taken using weighing scale (SECA® beam balance) calibrated before measurement and weight was recorded to nearest 10-g precision. The scale has a tare function that allows the weight of a cloth to be tared and then removed from the total weight. Thus, preterm weight was measured wrapped in warm cloth without a diaper, first the warm cloth was placed on the tray, then the (ON/TARE) button was pressed until the display showed “0:0.0”, then a preterm was wrapped on a warm cloth and placed on the scale and the displaced weight was recorded.

Research assistants were all thoroughly trained on measuring weight including how to operate the scale calibration and standardization, as well as how to document.

### Statistical analysis

Data analysis was performed using Statistical Package for Social Sciences (SPSS version 20.0) Descriptive statistics were used to summarize data as well as characterize the infants and mothers in the sample. The mean and standard deviation (SD), or median and interquartile range (IQR) were used to describe continuous variables, and percentages for categorical variables.

Independent variables were baseline characteristics, comorbid conditions, feeding practices and mother’s knowledge on preterm feeding. Based on responses on the five questions regarding mother’s knowledge, a summative score (5/5) was developed and categorized into low, moderate and high. (Low = less than 3/5, moderate =3/5 and high more than 3/5).

The outcome variable average weight gain (g/kg per day) was dichotomized into weight gain ≥15 g/kg/day (adequate weight gain) and weight gain < 15 g/kg/day (poor weight gain). To evaluate the weight change pattern a difference between subsequent weight and birth weight was calculated and plotted in a graph. Bivariate analysis was done to determine factors associated with poor weight gain in preterm infants. Pearson chi- square tests were used to compare distribution of categorical variables. Univariate logistic regression model was used to assess factors associated with poor weight gain in bivariate analysis, and factors with a *p*-value < 0.2 were entered in the multivariable logistic regression models to identify the independent predictors of poor weight gain. Odds ratio with 95% confidential intervals (CI) are presented, and statistically significance of results was determined at P- value < 0.05 and if 95% CI did not include 1.

### Ethical consideration

Ethical clearance was sought from the ethical committee of Institutional Review Board (IRB) of Muhimbili University of Health and Allied Sciences (MUHAS). Permission to conduct the study in the hospital was obtained from the ethical board of Muhimbili National Hospital (MNH/TRC/Permission/2018/402). Information about the study objectives its benefits and risks as well as a written consent was provided to the mothers/caretakers of eligible preterm prior to being recruited in the study. For mothers/caretakers who could not write, a verbal consent was obtained and a thumbprint was taken as a proof of the consent. The above consents and thumbprint procedure were approved by the ethical committee (IRB) of MUHAS. All information from patient was handled with maximum confidentiality. In case a preterm was found with poor weight gain, he/she was referred to the attending paediatrician for necessary intervention.

## Results

**Fig. 1:**
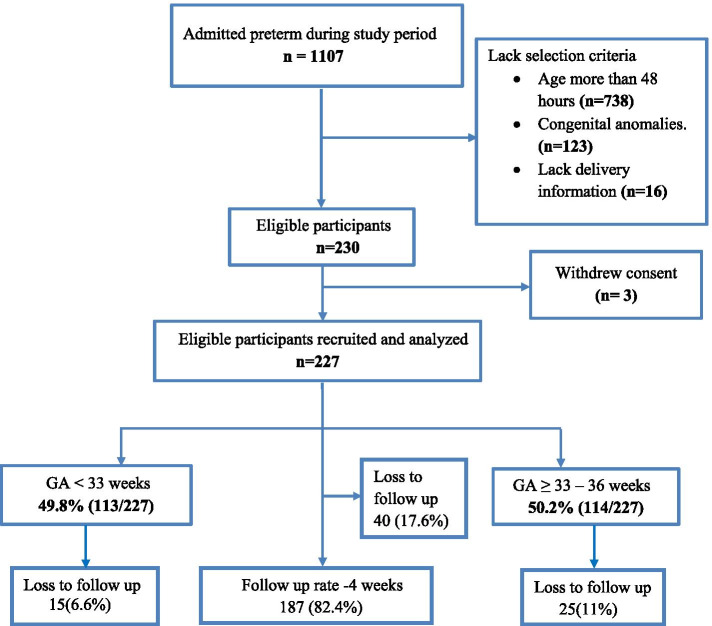
Flow diagram depicting flow of the study participants

A total of 1107 preterm neonates were admitted during the study period. Out of which 230 were selected and recruited into the study, consent was withdrawn for 3 participants therefore the final analysis included 227 participants. (Fig. 1).

Slight female preponderance of about (55.1%) with almost equal distribution in terms of GA among early and late preterm. The spread of weight amongst preterm were more on LBW (56.8%) than ELBW. Two-thirds of preterm (63.4%) were delivered by spontaneous vaginal delivery and higher proportion were born at MNH (74.9%). Majority of preterm (79.3%) had longer duration of hospital stay of 21–28 days. Both caretakers were aged between 18 and 30 years and more than half of mothers had primary school education (51.1%) (Table 1).

**Table 1:** Socio- demographics and baseline characteristics of preterm neonates and their caretakers

Variables	***N*** = 227	%
**Characteristics of the preterm neonate.**		
**Sex**		
Male	102	44.9
Female	125	55.1
**Birth Weight**^**a**^		
ELBW	21	9.3
VLBW	77	33.9
LBW	129	56.8
**Mode of delivery**^**b**^		
LSCS	83	36.6
SVD	144	63.4
**GA**		
Early preterm (< 33 weeks)	113	49.8
Late preterm (≥ 33–36 weeks)	114	50.2
**Duration of hospital stay, (days)**		
≤ 7	35	15.4
14–21	12	5.3
21–28	180	79.3
**Co-morbidity**		
Yes	108	47.6
No	119	52.4
**Neonatal Outcomes**		
Death	20	8.8
Discharged or alive at end of the study	207	91.2
**Place of delivery**		
MNH	170	74.9
Others	57	25.1
**Characteristics of the caretaker**		
**Age (mother)**		
18–30	149	65.6
30–45	75	33.0
46+	3	1.4
**Age (father)**		
18–30	109	48
31–45	104	45.8
46+	14	6.2
**Level of education (mother)**		
No formal education	10	4.4
Primary school	116	51.1
Secondary school	84	37.0
Post-secondary/university	17	7.5
**Level of education (father)**		
No formal education	3	1.3
Primary school	86	37.9
Secondary school	98	43.2
Post-secondary/university	40	17.6
**Occupation of mother**		
Housewife	90	39.7
Peasant	11	4.8
Government	7	3.1
Private sector	20	8.8
Self-employment	99	43.6
**Occupation of father**		
Peasant	6	2.7
Government	22	9.7
Private sector	40	17.6
Self-employment	159	70.0
**# of children mother has**		
One	75	33.0
Two	57	25.1
Three	47	20.7
> Three	48	21.2
Median (IQR)	2	(1, 3)
**Previous history of preterm**		
Yes	25	11.0
No	202	89.0

^a^Birth weight is categorized into extremely low birth weight (ELBW); Very low birth weight (VLBW) and Low birth weight (LBW)

^b^Mode of delivery is categorized into lower segment caesarean section (LSCS) and Spontaneous vaginal delivery (SVD)

**Fig. 2:**
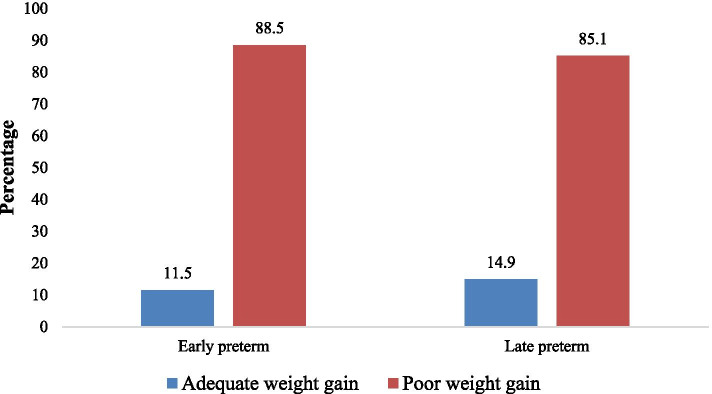
Percentage with growth deficit among early and late preterm

The average weight gain among preterm was 5.07 g/kg/day. Overall, 197/227 (86.8%) of the studied preterm neonates had poor weight gain (< 15 g/kg/day). Amongst those (88.5%) were early preterm neonates (Fig. 2).

**Fig. 3:**
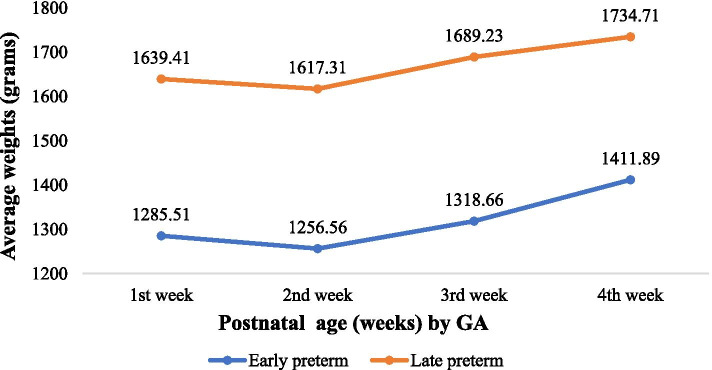
Trends of average weights with postnatal age (weeks) by GA

The overall trend of average weights of preterm neonates on a weekly basis during neonatal period showed varied loss of their body weights proportion in the 1st week of life depending on their maturity and regained birth weight from the 2nd week of life followed by gradual increase in weight**.** The average percentage weight loss in the first week of life was more (3.37%) in early preterm neonates. However, there was no significant difference in the proportion of their weight loss among the two categories (*p* = 0.38) (Fig. 3).

**Fig. 4:**
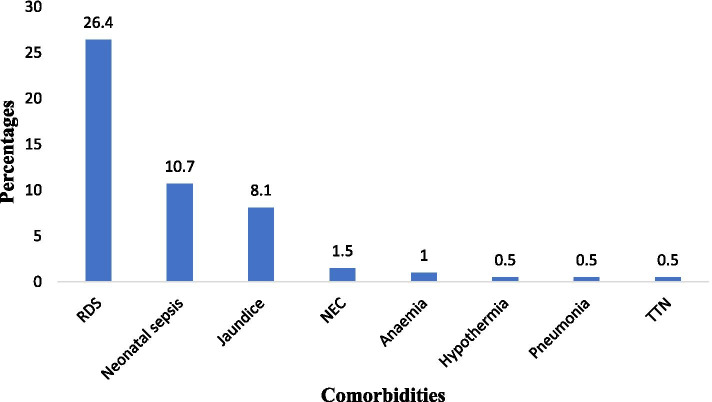
Co- morbidity conditions among study participants with poor weight gain

Among preterm neonates who had poor weight gain 97/197(49.2%) had multiple diagnoses i.e., more than one diagnosis. Common co-morbid conditions were RDS 25/97 (26.4%), neonatal sepsis 11/97 (10.7%) neonatal jaundice 8/97 (8.1%) and other diagnoses (Fig. 4).

As for the feeding practices, most of the preterm neonates initiated their first feed on second day of life, preferably breast milk, every 3 h with cup feeding being the main method of feeding (Supplementary Table 1.a).

With regard to feeding practices in association with weight gain, preterm who started on cup feeding upon admission and initiated first feed after 48 h had higher rate of poor weight gain (*p* = 0.028) and (*p*<0.001) respectively. Although, preterm that were fed every 3 h were likely to have poor weight gain compared to those fed every 2 h the difference was not statistically significant (Table 2).

**Table 2:** Feeding practices associated with weight gain among preterm neonates

Variable	Weight gain categories		
	Poor weight gain <15 g/1000 g/day	Adequate weight gain ≥ 15 g/1000 g/day	***P***- value
**Initial method of feeding**	**n (%)**	**n (%)**	
Cup	134(90.5)	14(9.5)	
Tube	55(82.1)	12(17.9)	
Breastfeeding	8(66.7)	4(33.0)	**0.028**
**Frequency of feeding**			
Every 2 h	16(80.0)	4(20.0)	
Every 3 h	181(87.4)	26(12.6)	0.31
**Time to initiation first feed**			
1st day	29(61.7)	18(38.3)	
2nd day and beyond	168(93.3)	12(6.7)	**< 0.001**
**Time to full feeds (days)**			
0–7	71(88.8)	9(11.3)	
8–14	79(84.9)	14(15.1)	
15–21	34(87.2)	5(12.8)	
22+	13(86.7)	2(13.3)	0.88
**Type of milk fed on admission**			
Breast milk	185(86.4)	29(13.6)	
Formula milk	4(100.0)	0(0.0)	
Mixed	8(88.9)	1(11.1)	0.86

Mothers were asked questions pertaining to routine feeding practices and the overall knowledge on feeding showed that poor weight gain was observed more among mothers with low knowledge (90.2%), compare to others, however, the difference was not significant (Table 3).

**Table 3:** Mother’s knowledge (response from mothers) in relation to weight gain

Variable	Weight gain categories		***P***-value
	Poor weight gain <15 g/1000 g/day	Adequate weight gain ≥ 15 g/1000 g/day	
**Ever received counselling on feeding. (*n*** **= 46)**	**n (%)**	**n (%)**	
Yes	39(84.8)	7(15.2)	0.80
No	158(87.3)	23(12.7)	
**Type of Milk preferable.**			
Breast milk.	181(86.6)	28(13.4)	*0.57
**Formula milk and mixed**	16(88.9)	2(11.1)	
**Optimal duration (EBF)**			
6 months	158(86.3)	25(13.7)	0.81
**< 6 months and I don’t know**	39(88.6)	5(11.4)	
**Preferable method for feeding**			
Breastfeeding	119(85.0)	21(15.0)	0.42
**Cup and tube**	78(89.7)	9(10.3)	
**Time after birth to initiate feeding.**			
First 24 h	117(85.4)	20(14.6)	0.55
**> 24 h and I don’t know**	80(88.9)	10(11.1)	
**Frequency of feeding.**			
Every 2 h	32(94.1)	2(5.9)	*0.27
**> 2 h and I don’t know**	165(85.5)	28(14.5)	
**Overall knowledge on feeding**			
Low	55(90.2)	6(9.8)	0.47
Moderate	67(88.2)	9(11.8)	
High	75(83.3)	15(16.7)	

Distribution of weight gain by clinical characteristics showed no statistically significant difference in weight gain between preterm neonates who had documented episodes of co-morbidities compared to those who did not have these complications (Table 4).

**Table 4:** Clinical characteristics on association with weight gain

Variable	Weight gain categories		***P***-value
	Poor weight gain <15 g/1000 g/day	Adequate weight gain ≥ 15 g/1000 g/day	
**RDS**	**n (%)**	**n (%)**	
Yes	52(81.2)	12(18.8)	0.64
No	145(89.0)	18(11.0)	
**Sepsis**			
Yes	21(91.3)	2(8.7)	*0.75
No	176(86.3)	28(13.7)	
**Jaundice**			
Yes	11(73.3)	4(26.7)	0.12
No	186(87.7)	26(12.3)	
**Other comorbidities**			
Yes	8(80.0)	2(20.0)	*0.63
No	189(87.1)	28(12.9)	
**Outcome**			
Death	19(95.0)	1(5.0)	*0.22
Discharged	178(86.0)	29(14.0)	
**Duration of hospital stay**			
≤ 14	31(88.6)	4(12.9)	0.21
15–21	8(66.7)	4(33.3)	
22–28	158(87.8)	22(12.2)	

^*^Fischer exact test

Following adjustment for confounders as shown in Table 5, predictors of poor weight gain were found to be low level of maternal education (AOR = 2.58; 95%Cl: 1.02–6.53), cup feeding as the initial method of feeding a preterm (AOR = 8.65; 95%Cl: 1.59–16.24) and delayed initiation of the first feed (AOR = 10.06; 95%Cl: 4.14–24.43). A previous history of preterm delivery appeared to be protective against poor weight gain (AOR = 0.33; 95% Cl: 0.11–0.79).

**Table 5:** Multivariable logistic regression of weight gain in relation to socio-demographic, feeding practices and clinical characteristics among studied preterm

Variable	COR (95% Cl)	AOR (95% Cl)	***p***-value
**Education (mother)**			
Primary/no formal education	2.42(1.09–5.36)	2.58(1.02–6.53)	**0.045**
Secondary and above	1	1	
**History of preterm delivery**			
Yes	0.33(0.13–0.88)	0.33(0.11–0.79)	**0.039**
No	1	1	
**Method of feeding**			
Cup	4.70(1.28–17.92)	8.65(1.59–16.24)	**0.001**
Tube	2.29(0.59–8.87)	1.99(0.369–10.73)	0.37
Breastfeeding	1	1	
**Time to initiation of first feed**			
First 24 h	1	1	
After 24 h	8.69(3.79–19.93)	10.06(4.14–24.43)	**< 0.001**
**Jaundice**			
Yes	0.38(0.11–1.30)	0.049(0.01–0.34)	0.20
No	1	1	

*CI* Confidence Interval, *COR* Crude Odds Ratio, *AOR* Adjusted Odds Ratio

## Discussion

The aim of this study was to determine the proportion of preterm with poor weight gain and its predictors among preterm neonates receiving care at MNH neonatal unit. We observed that the overall proportion of preterm with poor weight gain is 86.8%. The rates of postnatal poor weight gain were high among preterm during neonatal period with no significant difference between early and late preterm. The independent predictors of poor weight gain were found to be low maternal education level, delayed initiation of the first feed, cup feeding as an initial mode of feeding and history of prior preterm delivery. All these factors could have affected the nutritional intake, in these infants who have greater nutritional needs to match their expected high rates of growth.

The overall proportion of preterm with poor weight gain observed in this study was high. Furthermore the observed average weight gain is below the goal for preterm weight gain (≥ 15 g/kg/day) estimated to replicate the growth velocity of a normal foetus during third trimester of pregnancy [4, 11–14]. Poor weight gain was observed more among early preterm compared to late preterm infants, although this was not statistically significant, it may be a reflection of poor feeding as a result of feeding intolerance and also the practice in our unit during the study period of delaying initiation of feeds for at least 24 h for this category of neonates.

Several studies have reported similar findings of high proportion in poor weight gain among preterm [3, 12, 15–17], contrarily other studies have found marked advancement in the growth of the preterm [18, 19]. The difference observed across these studies in comparison to this study could be explained by the differences in characteristics of study participants, the different existing feeding protocols and the support available for supervising feeding of these vulnerable neonates. The observed high proportion of poor weight gain among preterm neonates in this study and other studies from similar setting could be attributed to various challenges such as low level of maternal education, inadequate staffing and co morbid conditions. Furthermore, in the studied setting the units are overcrowded, mothers are responsible for feeding their infants in presence of minimal supervision and time allocated for feeding may not be adequate, since rooming in is not feasible. Additionally, the differences can be explained by different study designs used. In prospective studies like the present one especially in areas where data is appropriately captured, the missing or incomplete data may not be appreciated hence result is well reported. The differences in sample size used and duration of follow up could also contribute to these variations in growth outcomes amongst preterm.

The study population consist of preterm neonates, categorized into early and late preterm. With regarding to the findings, the proportion of poor weight gain was highest among early preterm than the late preterm neonates, and the extent of poor weight gain was shown to increase with a decrease in gestation age and these has been supported by several studies [19, 20]. The implication of the above findings further observed a marked percentage weight loss in first week of life in early preterm followed by a pattern of a slow gradual increase until the 4th week postnatally. This can be explained by the fact that, the initial weight loss has been attributed to various reasons such as changes in body composition seen after birth which is more pronounced in preterm babies and suboptimal absorptive capacity of immature kidneys which favours water loss [20, 21]. Moreover, the immaturity in body organ systems leading to the absence of coordinated swallowing and sucking reflexes especially in preterm < 34 weeks GA poses difficulties with enteral feeding. These add to the pre-existing weight loss and hence contribute to postnatal growth failure among preterm. Furthermore, in presence of co-morbid conditions weight loss can be more and there may be delays in regaining birth weight.

Co-morbid conditions among preterm neonates are inevitable; due to prematurity of their body-organ systems. Preterm infants with co-morbid conditions are likely to experience poor weight than those without co-morbid conditions [22]. As revealed by our study no significant association between co-morbid conditions and poor weight gain was observed. Contrary to this study, several other studies have shown that co-morbid conditions in preterm are the compelling reasons for growth deficit [3, 19, 20]. Therefore, since co -morbidities in preterm are present due to prematurity; there is a biological plausibility to explain their influence on the weight pattern as observed in other studies above. There are possibilities that we did not capture all comorbid conditions as the diagnoses were mostly captured from the clinical notes. Thus, the few events captured did not achieve the adequate power to detect this association. Nevertheless, we cannot over emphasize the importance of early identification and treatment of comorbid complications. This has a key role in reduction of short- and long-term consequences including growth failure among preterm neonates.

Timing of initiation of enteral feeds has been shown to be a determinant of nutritional adequacy in preterm neonates. Delaying feeding (more than 48 h) is among the factors that have been reported in this study to increase the risk of poor weight gain. The association between late initiation of enteral feeding and poor growth in preterm was similarly observed in these studies [3, 17, 19, 23]. Additionally, other study showed that less aggressive progression of enteral feeds might increase the incidence of poor growth failure of both early and late preterm. Findings from all these studies emphasizes the relevance of the WHO recommendation for optimal feeding, that breastfeeding should be initiated early within 24 h of birth and infants unable to suck, breast milk to be given by other means until breastfeeding is possible [10]. Delayed initiation of the first feed regardless of the reason could also cause inadequate intake of nutrients and hence affect the growth rate.

In this study cup feeding, as an initial mode of feeding was associated with higher odds of poor weight gain. There are probabilities that, these neonates received inadequate amount of feeds as a result of some spilling [24] that could have occurred during feeding and thus contributing to inadequate intake of nutrients and hence poor weight gain. Our hospital has adapted the WHO recommendations, thus the protocol at MNH neonatal unit required early initiation of breast milk in neonates and for those in alternative oral feeding method to use a cup or a nasogastic/ orogastric tube in small volumes of feeds given frequently. However as shown in this study the feeding protocols were not strictly followed which could be a result of high rates of poor growth outcome among these preterm. This represents critical area where improvement is required to ensure responsive care to facilitate early initiation of feeds and appropriate feeding method to foster a healthy growth and development among preterm and low birth weight infants.

A prior history of preterm birth was less likely to be affected by poor weight gain. Although, there are limited studies that directly explain the influence of previous history of preterm and weight gain, there are possibilities that these mothers used their experience of caring for a preterm baby before. Thus, they had more knowledge that enabled them to complying with feeding recommendations that influenced a better feeding practice and hence adequate weight gain than those who had no prior exposure.

Low maternal education independently predicted poor weight gain among the preterm. There are probabilities that those with high level of education could understand better and retain the information provided during health talks related to neonatal feeding. Thus, they were more likely to have complied with established feeding protocols, consequently, less effect on quantity and frequency of feeds. Correspondingly, these studies [3, 12] reported the association of low maternal education and poor weight gain among preterm. Education always empowers mothers in all aspects and should therefore be emphasized across countries at large. Mothers, who lack or have very low level of literacy, should therefore be given extra support and supervision during feeding to assist them in caring for their preterm babies. Special attention is required to ensure adequate feeding, proper caretakers counselling and early evaluation in all preterm neonates to optimize their growth. Future studies are needed to determine other predictors of poor weight gain among preterm neonates that were beyond the scope of this study.

Preterm neonates are at risk of growth deficits during hospitalization, thus strategies to strengthen the provision of health care services must be in place. This should include modification of existing guidelines to strengthen consistent monitoring of weights, early identification and treatment of comorbidities. This will empower clinicians and nurses with knowledge necessary to identify preterm at risk of poor growth and provide immediate measures.

Existing protocol on time to initiate first feed to preterm, within 24 h (1st hour of age) should be emphasized, to reduce the magnitude of poor postnatal growth. A close supervision should be provided to ensure adequate feeding for preterm on cup feeding, and where indicated, tube feeding should be used, especially for preterm and sick neonates unable to breastfeed. Health education and counselling to caretakers should focus on recommended feeding practices and optimal growth of preterm. This should target mothers with low education level to help them abide to the basics in taking care of their neonates by giving them extra support, discharging their preterm at a much higher weight or even giving them close follow up of 1–2 weeks postnatal return date to neonatal clinic. More studies with a larger cohort involving more study sites and longer duration would be more appropriate to improve the generalizability of the results.

Our study findings should be interpreted bearing the following limitations in mind. The estimated GA may not always be accurate since some of them were based on last normal menstrual period of the mothers/ Ballard score. Foetal ultrasonography is the most accurate technique for estimating gestational age in antenatal clinic, however it is not routinely done and most mothers cannot afford. We were not able to use a dry weight since it was not feasible to starve these neonates. Challenges when measuring weight for neonates who were very sick and kept on CPAP. However, the weights were measured using a calibrated scale and was found to be consistent after being weaned off CPAP. The recorded information was obtained from the mothers and some from the clinical files such as co-morbid conditions hence recall bias and incomplete documentation respectively. This could have resulted in underestimation of these co-morbidities and hence lack of adequate power to detect the true difference between co-morbid condition and poor weight gain.

Furthermore, the knowledge score used in this study was self- created based on modified questions from WHO’s guidelines on optimal feeding of a preterm [10]. However, these were normal questions derived from basic information recommended by WHO to be used for health education given to the mothers during breastfeeding sessions.

## Conclusion

The proportion of poor weight gain among preterm neonates admitted at MNH was high. Early preterm have high proportion of poor weight gain with slight increase in percentage weight loss in the first week of life than late preterm. Predictors of poor weight gain among preterm infants were delayed initiation of feeds, cup feeding as initial method of feeding and low education level of mothers. On the other hand, a previous history of preterm delivery appeared to be protective against poor weight gain.

## Supplementary Information

**Additional file 1: Supplementary Table 1(a).** Attributes of feeding practices among preterm neonates. **Supplementary Table 1(b).** Caretaker’s knowledge on feeding practices. **Supplementary Table 1 (c).** Neonatal feeding advancement guideline. (MUHIMBILI NEONATAL UNIT PROTOCOL).

## Data Availability

The data set supporting the conclusion of this article is available from corresponding author on reasonable request.
