# Supplementary material for: Poor weight gain and its predictors among preterm neonates admitted at Muhimbili National Hospital in Dar-es-salaam, Tanzania: a prospective cohort study
Source: BMC Pediatr. 2021 Nov 6;21:493. doi: 10.1186/s12887-021-02971-y (PMC8571859; doi:10.1186/s12887-021-02971-y)
Supplement: Supplementary file 1 — Additional file 1: Supplementary Table 1(a). Attributes of feeding practices among preterm neonates. Supplementary Table 1(b). Caretaker’s knowledge on feeding practices. Supplementary Table 1 (c). Neonatal feeding advancement guideline. (MUHIMBILI NEONATAL UNIT PROTOCOL). [file 12887_2021_2971_MOESM1_ESM.docx]

**TITLE: Poor weight gain and its predictors among preterm neonates admitted at Muhimbili National hospital in Dar-es-salaam, Tanzania: A Prospective Cohort Study.**

**Authors**

Victoria Paul Ndembo^1^, Helga Naburi^2^, Rodrick Kisenge^2^, Germana H. Leyna^3^, Candida Moshiro^3^

## Supplementary Table 1(a). Attributes of feeding practices among preterm neonates

| **Variables.** | **N** | **%** |
| --- | --- | --- |
| **Method of feeding initiated** |  |  |
| Cup | 122 | 53.7 |
| Tube | 77 | 33.9 |
| Breastfeeding | 28 | 12.4 |
|  |  |  |
| **Frequency of feeding** |  |  |
| Every 2 hours | 20 | 10.1 |
| Every 3 hours | 207 | 89.9 |
|  |  |  |
| **Time to initiation of first feed** |  |  |
| 1^st^ day of life | 33 | 14.5 |
| 2^nd^ day of life | 122 | 53.8 |
| 3^rd^ day of life | 55 | 24.2 |
| >3 days of life | 17 | 7.5 |
|  |  |  |
| **Time to full feeds (days)** |  |  |
| 0-7 | 80 | 35.2 |
| 8-14 | 93 | 41.0 |
| 15-21 | 39 | 17.2 |
| 22+ | 15 | 6.6 |
| **Type of milk fed upon admission** |  |  |
| Breast milk | 214 | 94.3 |
| Formula milk | 4 | 1.8 |
| Mixed | 9 | 3.9 |

## Supplementary Table 1(b). Caretaker’s knowledge on feeding practices.

| **Variables.** | **N** | **%** |
| --- | --- | --- |
| **Ever received counselling on feeding** |  |  |
| Yes | 46 | 20.2 |
| No | 181 | 79.8 |
| **Place mother received the counselling (n=46)** |  |  |
| During ANC | 1 | 2.2 |
| Delivery Hospital | 3 | 6.5 |
| Neonatal Unit | 39 | 84.8 |
| Home | 3 | 6.5 |
| **Provider of counselling (n=46)** |  |  |
| Doctor | 3 | 6.5 |
| Nurse | 41 | 89.1 |
| Other  **Knowledge variables.** | 2 | 4.4 |
| **Type of Milk preferable for feeding** |  |  |
| Breast milk | 192 | 84.6 |
| Formula | 1 | 0.4 |
| Don’t know | 34 | 15.0 |
| **Optimal duration of EBF** |  |  |
| 3 months | 1 | 0.4 |
| 4 months | 2 | 0.9 |
| 6 months | 183 | 80.6 |
| Don’t know | 41 | 18.1 |
| **Preferred method for feeding** |  |  |
| Cup | 56 | 24.7 |
| Tube | 28 | 12.3 |
| Breastfeeding | 143 | 63.0 |
| **Time after birth to initiate feeding** |  |  |
| First 24 hours | 139 | 61.2 |
| 48 hours | 35 | 15.4 |
| 72 hours | 5 | 2.2 |
| Don’t know | 48 | 21.2 |
| **Frequency of feeding** |  |  |
| Every 2 hours | 34 | 15.0 |
| Every 3 hours | 160 | 70.5 |
| Every 4 hours | 3 | 1.3 |
| Don’t know  **Knowledge on feeding**  Low  Moderate  High | 30  61  76  90 | 13.2  26.9  33.5  39.6 |

*EBF- Exclusive breast-feeding*

## Supplementary Table 1 (c). Neonatal feeding advancement guideline. (MUHIMBILI NEONATAL UNIT PROTOCOL).

| DAYS | Fluid and enteral feeds/ 24 hours |  | Type of IV fluid. |
| --- | --- | --- | --- |
| Day 1 | 80mls/kg, PO - colostrum swab |  | D10 |
| Day 2 | IV 100mls/kg, PO 10mls/kg |  | D10 |
| Day 3 | IV 100mls/kg, PO 30mls/kg |  | D10+1/4 NS (80mlsD10+20mlsNS) |
| Day 4 | IV100mls/kg, PO 50mls/kg |  | D10+1/4 NS |
| Day 5 | IV 80mls/kg, PO 70mls/kg |  | D10+1/4 NS |
| Day 6 | IV60mls/kg, PO 100mls/kg |  | D10+1/4 NS  Disconnect IV fluids if the baby is ok |
| Day 7 | PO 130mls/kg |  | No IV fluids |
| Day 8 | PO 160mls/kg |  | No IV fluids |

*PO – per oral feeding (refers to breast milk or formula), IV- Intravenous fluids, NS- normal saline, D10- dextrose 10% concentration*
